# Do we know enough about the effect of low-dose computed tomography screening for lung cancer on mortality to act? An updated systematic review, meta-analysis and network meta-analysis of randomised controlled trials 2017 to 2021

**DOI:** 10.1186/s41512-023-00162-0

**Published:** 2023-12-11

**Authors:** Emma Duer, Huiqin Yang, Sophie Robinson, Bogdan Grigore, Josie Sandercock, Tristan Snowsill, Ed Griffin, Jaime Peters, Chris Hyde

**Affiliations:** 1 Queen Mary University of London, Barts and The London School of Medicine and Dentistry, London, UK; 2 School of Healthcare, Faculty of Medicine & Health, University of Leeds, Leeds, UK; 3 Evidence Synthesis & Modelling for Health Improvement, University of Exeter Medical School, Exeter, UK; 4 Exeter Test Group, University of Exeter Medical School, Room 1.22 College House, St Luke’s Campus, Exeter, EX1 2LU UK; 5 Systematic Review Initiative, NHS Blood & Transplant, Oxford, UK; 6 Health Economics Group, University of Exeter Medical School, Exeter, UK

**Keywords:** Lung neoplasms, Mass screening, Early detection of cancer, Tomography, X-ray computed tomography, Spiral computed, Review, Systematic

## Abstract

**Background:**

For people at high risk of lung cancer, low-dose computed tomography (LDCT) is proposed as a method to reduce mortality.

**Methods:**

Our objective was to estimate the effect of LDCT lung cancer screening on mortality in high-risk populations.

A systematic review of randomised controlled trials (RCTs) comparing LDCT screening programmes with usual care (no screening) or other imaging screening programme (such as chest X-ray (CXR)) was conducted. RCTs of CXR screening were additionally included in the network meta-analyses. Bibliographic sources including MEDLINE, Embase, Web of Science and the Cochrane Library were searched to January 2017, and then further extended to November 2021. All key review steps were done by two persons. Quality assessment used the Cochrane Risk of Bias tool. Meta-analyses were performed.

**Results:**

Nine RCTs, with up to 12.3 years of follow-up from randomisation, were included in the direct meta-analysis, which showed that LDCT screening was associated with a statistically significant decrease in lung cancer mortality (pooled relative risk (RR) 0.86, 95% confidence interval [CI] 0.77 to 0.96). There was a statistically non-significant decrease in all-cause mortality (pooled RR 0.98, 95% CI 0.95 to 1.01). The statistical heterogeneity for both outcomes was minimal. Network meta-analysis including the nine RCTs in the direct meta-analysis plus two further RCTs comparing CXR with usual care confirmed the size of the effect of LDCT on lung cancer mortality and that this was very similar irrespective of whether the comparator was usual care or CXR screening.

**Conclusions:**

LDCT screening is effective in reducing lung cancer mortality in high-risk populations. The uncertainty of its effect on lung cancer mortality observed in 2018 has been much reduced with new trial results and updates to existing trials, emphasising the importance of updating systematic reviews. Although there are still a number of RCTs unreported or in progress, we predict that further evolution of summary mortality estimates is unlikely. The focus for debate now moves to resolving uncertainty about the cost-effectiveness of LDCT screening taking into account the balance between benefits and harms which occur in all screening programmes.

**Supplementary Information:**

The online version contains supplementary material available at 10.1186/s41512-023-00162-0.

## Introduction

The worldwide burden of lung cancer is huge. Of approximately 20 million cancer cases and 10 million cancer deaths estimated in 2020, lung cancer accounted for 11.4% of cases (2.2 million) and 18% of deaths (1.8 million). The burden is large irrespective of gender or a country’s development status [1]. It is not improving over time [2], and in some parts of the world may still be worsening [3]. Outcome is poor even in the best-performing countries, with 5-year survival being in the range of 10–30% without marked differences between more and less developed countries. Improvement over time seems modest, 5% over the period 2000 to 2014 in many countries [4]. Late presentation at advanced stage is a consistent feature of lung cancer, as is markedly improved survival at early stage [5]. Data on the latter are much more sparse in less-developed countries, but data from India suggest that improved survival at earlier stage probably holds generally [6].

Differential outcome by stage suggests that screening might be an approach to reducing mortality. Low-dose computed tomography (LDCT) has emerged as the strongest candidate for a screening test to achieve earlier detection of asymptomatic lung cancer and stage shift [7]. A number of countries, like the USA, have already introduced screening based on early evidence. Many more, such as Australia and the UK, are in the process of introducing it. However, widespread caution remains in other countries partly because of the need for empirical verification of effectiveness and the complexity of this evidence. The scale and cost of introduction are undoubtedly other important barriers to implementation. Concerning effectiveness, well-recognised challenges to assessing screening programmes like lead time bias, length bias and overdiagnosis mean that this should ideally be done by randomised controlled trials (RCT) measuring disease specific and all-cause mortality [8–10]. A further challenge for preventing lung cancer is that the main aetiological factor for lung cancer, cigarette smoking, also predisposes to other potentially fatal diseases particularly respiratory and cardiovascular diseases and other cancers, to which an individual may succumb if lung cancer is avoided [11]. Screening for lung cancer would be targeted at those at high risk, unlike other screening programmes which are offered to all persons of a given age and gender.

In 2018, our research group reported a systematic review and network meta-analysis of RCTs of LDCT screening for lung cancer with particular focus on the effect on disease-specific and all-cause mortality, searching up to 2017 [12, 13]. We identified 12 eligible RCTs of which four contributed data to the direct meta-analyses of LDCT vs CXR or usual care, the remainder being on-going studies. The summary estimates for LDCT screening against usual care in studies with up to 9.80 years of follow-up demonstrated a statistically non-significant decrease in lung cancer mortality (pooled relative risk (RR) 0.94, 95% CI 0.74 to 1.19) and a statistically non-significant increase in all-cause mortality (pooled RR 1.01, 95% CI 0.87 to 1.16). The estimated RR for lung cancer mortality in the network meta-analysis was 0.95 (95% CI 0.82 to 1.11). There was considerable uncertainty arising from the largest of the RCTs comparing LDCT with CXR screening rather than no screening, imprecision of the summary estimates, and important heterogeneity between the included study results. We suggested that maturing trials would be expected to resolve uncertainty and that decisions should be delayed until the results were available. This view was consistent with other systematic reviews [7, 14], but there were other calls for immediate action implying that the evidence was already adequate [15, 16].

Here we report an update to our systematic review and meta-analyses. Like the original systematic review, the update was commissioned to support decision making by the UK National Screening Committee.

## Methods

Our objective was to evaluate the clinical effectiveness of screening programmes for lung cancers with LDCT in high-risk populations using a systematic review, meta-analysis, and network meta-analysis of RCTs. The wider project also considered cost-effectiveness. The original systematic review and its update was registered (PROSPERO CRD42016048530). All aspects of the update were undertaken in accordance with the original pre-specified protocol with some minor recorded exceptions [17]. These involved an expansion of the range of outcomes we abstracted data on, searching some different websites to those originally specified and being more precise about what constituted poor study quality in the investigation of heterogeneity.

We extended our original search of MEDLINE, MEDLINE In-Process, Embase, PsycINFO (all via Ovid), Web of Science (Thomson Reuters), CDSR and CENTRAL (via The Cochrane Library), and CINAHL (EBSCO) from October 2016 to November 2021 (Web Table 1. MEDLINE search strategy). Literature prior to 2004 was identified via the 2006 health technology assessment by Aberdeen Health Technology Assessment Group [18] and literature from 2004 to 2017 was identified from our original systematic review [12]. Other published literature in the update was identified from reference checking of relevant systematic reviews.

In the main systematic review and meta-analysis, we included LDCT lung cancer screening programme RCTs involving populations at high risk of lung cancer. Any definition of high risk was eligible. LDCT screening programmes included both single and multiple rounds. The eligible comparators were no screening or other imaging technology screening programmes (such as CXR). RCTs evaluating the effectiveness of CXR but not LDCT were also included in the network meta-analysis. The outcomes of interest were lung cancer mortality and all-cause mortality, with only lung cancer mortality considered for the network meta-analysis because of insufficient data to construct a network for all-cause mortality.

Two researchers independently screened the titles and abstracts of all reports identified by the search strategy. Full-text papers were subsequently obtained and screened in the same way. Data extraction and quality assessment were undertaken by one researcher and checked by a second. The risk of bias of included studies was assessed using the Cochrane Risk of Bias tool [19]. We also considered underpowered sample size for important outcomes and substantial baseline differences between study arms on important characteristics.

All data were tabulated and primarily considered in a narrative review. DerSimonian and Laird random effect model meta-analyses were used to pool the estimates of effect [20]. We restricted the meta-analysis to RCTs with at least 5 years follow-up consistent with the primary outcome in the National Lung Screening Trial (NLST). The result for the longest period of follow-up was used. A random effects approach was pre-specified as part of the protocol development process; a fixed effects (or common effect) model was not favoured as it was thought highly unlikely that chance alone would account for differences between the results of included studies. Statistical heterogeneity was assessed using τ^2^ and the *I*^2^ statistic. Based on the advice in the Cochrane handbook, 30 to 50% was categorised as moderate heterogeneity and 50% upwards as substantial heterogeneity [21]. We considered the following factors for the exploration of heterogeneity, if present: quality of trials (particularly adequacy of randomisation), nature of interventions (e.g. frequency of LDCT screening), and nature of control groups (e.g. best available care such as CXR screening or usual care).

Network meta-analysis was performed to assess the relative effectiveness of three screening strategies (LDCT, CXR, and usual care). The original review used mvmeta in Stata [22]. For the update, we used netmeta, a comparable package in R [23, 24]. Both packages use a frequentist approach to network meta-analysis and implement similar methods for calculating ranking probabilities [25]. The original data were analysed using the R code for this update to check that results were consistent with mvmeta for this dataset. Direct and indirect evidence were plotted to assess the presence of inconsistency.

## Results

**Fig. 1:**
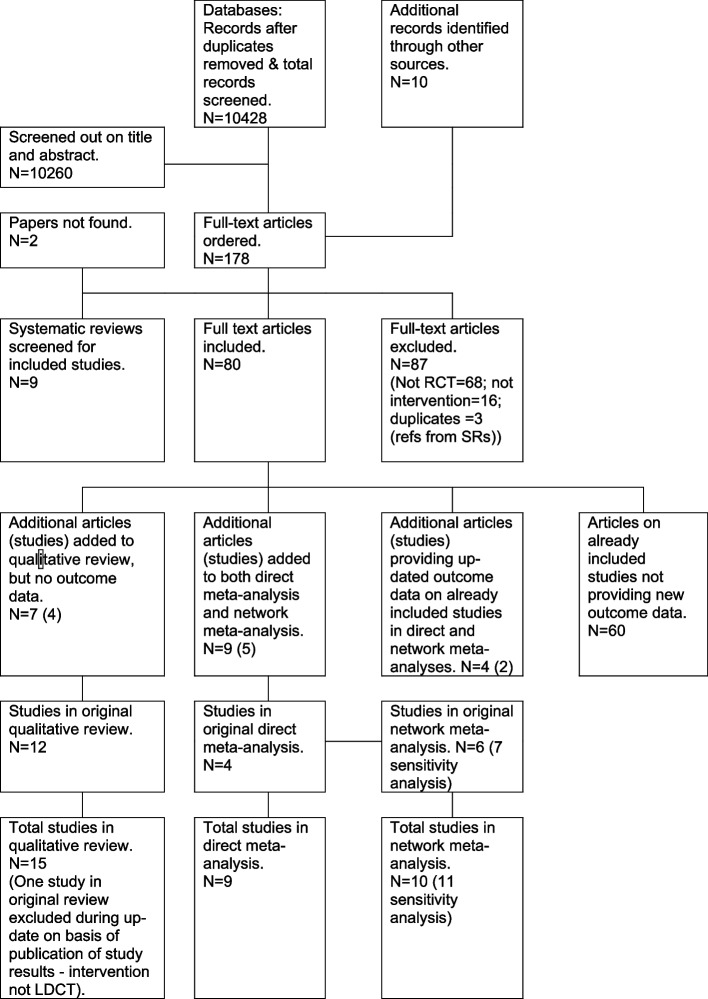
PRISMA diagram

In total, 10,428 records were screened. From these, 178 full texts were assessed for eligibility, from which 80 articles were included. These comprised seven articles referring to four new RCTs not identified in the previous review, and not providing outcome data to allow inclusion in the meta-analyses [26–29]; nine articles referring to five RCTs previously identified, and providing new data to allow inclusion in the meta-analyses [30–35]; four articles referring to two RCTs previously included in the meta-analyses and providing updated data on outcomes [36–38]. The remaining included articles were of previously included RCTs but not providing new data on the outcomes of interest. The large number of these indicates the great multiplicity of publications arising from each RCT. The disposition of the results of the search are further summarised in Fig. 1.

In combination with the included studies in the original systematic review, there were 15 RCTs included in the qualitative systematic review [27–30, 35–52] (Web Table 2). One RCT included in the original review was excluded during re-examination on the basis of the final results as part of the update which clarified that the screening intervention was not LDCT alone [53]. In total, there were nine RCTs included in the direct meta-analyses comparing LDCT to CXR or usual care [31–36, 38, 49, 50, 54, 55], two RCTs comparing CXR with usual care [56–61] included in the base-case network meta-analysis and one more included in a sensitivity analysis [62].

The characteristics of the studies included in the direct and network meta-analyses are shown in Table 1. The LDCT trials were all conducted in Europe and the USA. There was great variation in sample size from 2811 randomised to 53,454. The participants were aged from 49 to 75 years, were all high risk by virtue of being smokers or ex-smokers, and were all volunteers. There was a predominance of male participants particularly in the case of Detection and Screening of Early Lung Cancer with Novel Imaging Technology and Molecular Essays (DANTE) [39], Nederlands Leuvens Longkanker Screenings Onderzoek (NELSON) [47] and UK Lung Screening Trial (UKLS) [52]. There was some variation between the LDCT programmes, but typically they involved 4 or 5 rounds of LDCT screening over 4 to 6.5 years and which were compared to no screening. UKLS [52] was a pilot with only one round of screening. Where stated both study arms were offered smoking cessation. Of the trials, NLST [49] stands apart, not just in terms of large size, with over 50,000 participants, but by LDCT being compared to CXR screening rather than no screening and there being just three screening rounds. There was one other small study comparing two LDCT screens with CXR [44], this having acted as a pilot for NLST. All studies provided over 5 years of follow-up from randomisation, typically 10 years. However, in many trials because of the long duration of the interventions, the follow-up was less than 5 years after completion of screening (as opposed to follow-up from the start of screening just after randomisation) (DANTE [39], German lung cancer screening intervention (LUSI) [45], Lung Screening Study (LSS) [44], Multicentric Italian Lung Detection (MILD) [46], NELSON [47]).

**Table 1:** Characteristics of included studies

Study/country	Recruitment time	Screening programme	Comparator	Size (n)	Age (yrs) Gender (% male)	Entry	Number of screening rounds	Screening times and interval (yrs)	Duration of follow-up (mean/ median)
Direct meta-analysis and network meta-analysis – RCTs of LDCT screening									
DANTE [39] Italy	03/2001 to 02/2006	LDCT, medical exam and one CXR	No screening, medical exam and one CXR	2811	60–74 100	Volunteers Smokers & ex-smokers	5 vs 0	T0, T1, T2, T3, T4	At 12/2012, median 6 yrs 3.5 months
DLCST [41] Denmark	10/2004 to 03/2006	LDCT	No screening	4104	50–70 55	Volunteers Smokers & ex-smokers	5 vs 0	T0, T1, T2, T3, T4	At 4/2015, median 9.8 yrs both arms
ITALUNG [43] Italy	01/2004 to 12/2006	LDCT, smoking cessation programme	No screening, smoking cessation programme	3206	55–59 65	Volunteers Smokers & ex-smokers	4 vs 0	T0, T1, T2, T3	At 12/2014, median 9.3 yrs At 10/2019 ^a^, median 11.3 yrs
LSS [44] USA	Randomised 9/2000 to 01/2001	LDCT	CXR	3318	55–74 59	Volunteers Smokers and ex-smokers	2 vs 2	T0, T1	At 12/2005, median 5.2 yrs both groups
LUSI [45] Germany	09/2007 to 04/2011	LDCT, smoking counselling	No screening, smoking counselling	4052	50–69 65	Volunteers Smokers and ex-smokers	5 vs 0	T0, T1, T2, T3, T4	At 04/2018, mean 8.8 yrs overall
MILD [36, 46] Italy	09/2005 to 09/2011	LDCT (annual and biannual), smoking cessation, pulmonary function test, blood sample	No screening, smoking cessation, pulmonary function test, blood sample	4099	> 49 66	Volunteers Smokers and ex-smokers	7 or 4 vs 0	T0, T1, T2, T3, T4, T5, T6 vs T0, T2, T4, T6 ^b^	At 01/2011, median 4.4 years, both arms At 06/2018, “10 year results”
NELSON [47, 48] The Netherlands & Belgium	07/2003 to 12/2006	LDCT	No screening	15,822	50–75 84	Volunteers Smokers and ex-smokers	4 vs 0	T0, T1, T3, T5.5	At 12/2015, “minimum follow-up of 10 years”
NLST [49–51] USA	08/2002 to 04/2004	LDCT	CXR	53,454	55–74 59	Volunteers Smokers and ex-smokers	3 vs 3	T0, T1, T2	At 12/2009, median 6.5 yrs both groups At 07/2018 ^a^, median 12.3 yrs
UKLS [35, 52] UK	10/2011 to 02/2013	LDCT	No screening	3968	50–75 75	Volunteers High risk based on LLPv2	1 vs 0	T0	At September 2021: median 7.3 years
Network meta-analysis (main)—RCTs of CXR screening									
Czech [56, 57] Czech republic	06/1976 to 06/1977	Intensive CXR, sputum cytology	Single CXR, sputum cytology	6346	40–64 100	Non-volunteers Smokers	6 vs 1	T0, T0.5, T1, T1.5, T2, T2.5, T3 vs T0, T3 Further follow-up CXRs (no sputum) T4, T5,T6 in both arms	At 6/2000 ^a^, “Year 15 since enrollment”
MAYO [59, 60] USA	11/1971 to 07/1976	Intensive CXR, sputum cytology	Usual care (recommended an annual CXR and sputum cytology)	9211	> 45 100	Non-volunteers Smokers	18 vs unknown	T0.3, T0,7, T1, T1.3, T1.7, T2.0, T 2.3, T2.7, T3, T3.3, T3.7, T4, T4.3, T4.7, T5, T5.3, T5.7, T 6 (4 monthly) vs unknown	At 12/1996, median 20.5 yrs
Network meta-analysis (sensitivity)—RCTs of CXR screening (post hoc defined high-risk sub-group of larger RCT)									
PLCO [62] USA	1993 to 2001	CXR	No screening	154,901 (30,321 NLST eligible subgroup)	55–74 61	Volunteers Smokers and ex-smokers	4 vs 0	T0, T1, T2, T3	At 12/2009, 6 yrs all participants NLST eligible subgroup. 13 yrs results “similar”

*Abbreviations*: *CXR* chest X-ray, *LDCT* low-dose computed tomography, *RCT* randomised controlled trial, *N* not reported, *yrs* years

^a^Submission date of article reporting results

^b^Based on median duration of screening of 6.2 years

The two additional trials for the network meta-analysis compared intensive screening with CXR and sputum cytology over 3 to 6 years with usual care involving occasional CXR examination [56, 59, 60]. The frequency of screening in the intervention arms was much more frequent than the LDCT RCTs, with CXR examinations two or three times a year. The RCTs were done in the Czech Republic and USA in the 1970s with long follow-up. The participants were smokers, aged between 40 and 70 years, were exclusively male, and were non-volunteers. A third RCT of CXR screening conducted in the USA in the 1990s, Prostate, Lung, Colorectal and Ovarian cancer screening trial (PLCO) [62], could not be included because the majority of subjects were low risk. We did however include a post hoc high-risk sub-group analysis of this trial in a sensitivity analysis as this subgroup (NLST-eligible subgroup involving high-risk participants) was relevant to our research question. It compared four annual rounds of CXR screening with no screening.

As shown in Table 2, with notes on the justification of risk of bias assessments in Web Table 3, the majority of the LDCT included trials were judged to be of moderate to high quality overall, although allocation concealment was consistently poorly addressed, except in the cases of Italian lung cancer screening (ITALUNG) [43] LSS [44] and UKLS [52]. Random sequence generation, blinding of outcome assessment, complete outcome data collection and avoidance of selective reporting were strong features. None of the studies had blinding of participants and personnel, but they were still deemed to be at low risk of bias because of the objective nature of the mortality outcomes. Further protection against performance bias may have been afforded by active comparator arms in NLST [49] and LSS [44]. One RCT, MILD [46], was judged to be of much poorer quality than the other included studies with a particularly marked risk of bias arising from lack of clarity about randomisation, accompanied by marked imbalances in some of the baseline characteristics, particularly for the comparison of LDCT versus no screening. The imbalance was not the case for other trials, including those where there was also lack of clarity about randomisation method [34, 59, 60] (Web Table 4).

**Table 2:** Quality assessment of included studies

Study	Random sequence generation (selection bias)	Allocation concealment (selection bias)	Blinding of participants and personnel (performance bias)	Blinding outcome assessment (detection bias)	Incomplete outcome data (attrition bias)	Selective reporting (reporting bias)	Other risk of bias (power & baseline imbalance)
Direct meta-analysis and network meta-analysis—RCTs of LDCT screening							
DANTE [39]	Low	Unclear	Low	Low	Low	Low	Low & low
DLCST [41]	Low	Unclear	Low	Low	Low	Low	Low & low
ITALUNG [43]	Low	Low	Low	Low	Low	Low	Unclear and low
LSS [44]	Low	Low	Low	Low	Low	Unclear	High and low
LUSI [45]	Low	Unclear	Low	Low	Low	Low	Unclear and low
MILD [46]	Unclear	Unclear	Low	Low	Low	Low	High and high
NELSON [47]	Unclear	Unclear	Low	Low	Low	Low	Low and low
NLST [49]	Low	Unclear	Low	Low	Low	Low	Low and low
UKLS [52]	Low	Low	Low	Low	Low	Low	Unclear and low
Network meta-analysis (main) – RCTs of CXR screening							
Czech [56]	Low	Unclear	Low	Unclear	Unclear	Low	Unclear and low
MAYO [59, 60]	Unclear	Unclear	Low	Low	Low	Low	Low and low
Network meta-analysis (sensitivity)—RCTs of CXR screening (post hoc defined high-risk sub-group of larger RCT)							
PLCO [62]	Low	Unclear	Low	Low	Low	Low	High and low

*Abbreviations*: *CXR* chest X-ray, *LDCT* low-dose computed tomography, *RCT* randomised controlled trial, *N* not reported, *yrs* years

Descriptors for each aspect of study quality indicate risk of bias

The additional RCTs of CXR vs no screening, included for the network meta-analysis only, were of slightly poorer methodological quality than most of the LDCT RCTs, with less clarity about loss to follow-up and absence of power calculations. A mitigating factor may be that standards for reporting RCTs were not well established in the 1970s when the studies were conducted with the first Consolidated Statement of Reporting of Trials version being published in 1996 [63]. Although the PLCO main trial [62] was of similar quality to the LDCT RCTs, the NLST sub-group study admitted very limited power to detect small differences in mortality and was only able to demonstrate baseline equivalence for a small number of characteristics.

**Fig. 2:**
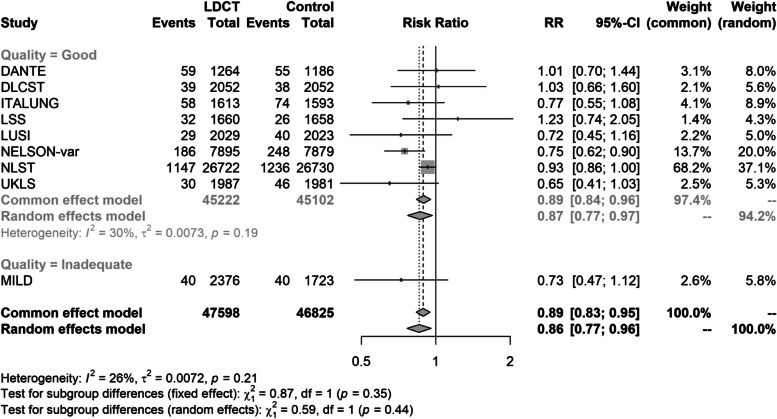
Lung cancer mortality—results

The direct meta-analysis showed that LDCT screening is associated with a statistically significant decrease in lung cancer mortality (pooled RR 0.86, 95% CI 0.77 to 0.96; *p* = 0.007) with follow-up ranging from 5.2 to 12.3 years from randomisation when compared with controls (Fig. 2) and little statistical heterogeneity in the magnitude of effects (*I*^2^ = 26%, τ^2^ = 0.0072, *p* = 0.21%). Sources of heterogeneity were not investigated, but removing the poorest-quality trial (MILD) [36] in a sensitivity analysis made no substantive difference to the results (pooled RR 0.87, 95% CI 0.77 to 0.97; *p* = 0.018). With little heterogeneity, results for the fixed (common) effects model were not substantially different than those from the random effects model (Fig. 2).

**Fig. 3:**
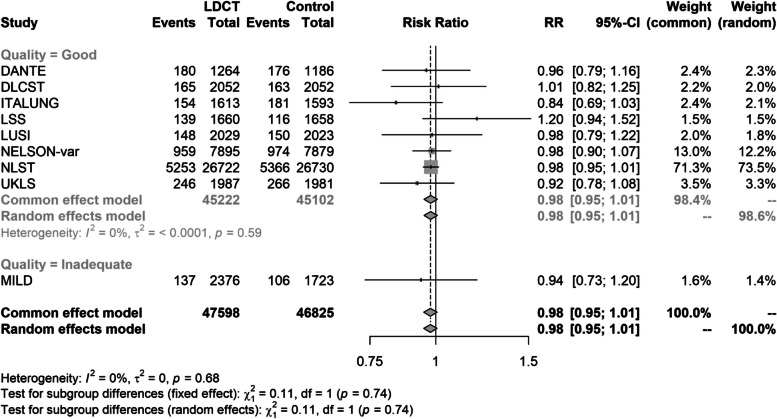
All-cause mortality—results

There was a statistically non-significant reduction in all-cause mortality compared with controls (pooled RR 0.98, 95% CI 0.95 to 1.01; *p* = 0.19) (Fig. 3). The follow-up ranged from 5.2 to 12.3 years, as for lung cancer mortality, with the exception of ITALUNG [31, 32] where all-cause mortality was only available at a median of 9.3 years, in contrast to a median of 11.3 years for lung cancer mortality. The level of statistical heterogeneity was again low (*I*^2^ = 0%). The sensitivity analysis removing the low-quality MILD study made no difference to the summary estimate (pooled RR 0.98, 95% CI 0.95 to 1.01).

Network meta-analysis assessed the relative effectiveness of LDCT, CXR screening, and usual care with respect to lung cancer mortality, data for all-cause mortality not being available for all parts of the network. The main network consisted of six RCTs comparing LDCT with usual care [31, 33, 34, 36, 54, 55]; two trials comparing LDCT with CXR [35, 38]; and two trials comparing CXR with usual care [58, 61]. A further RCT of CXR vs usual care (PLCO) was included in a sensitivity analysis [62]. In the main network meta-analysis, the estimated RR of lung cancer mortality of LDCT compared to usual care was 0.86 (95% CI 0.75 to 0.98), of LDCT compared to CXR 0.85 (95% CI 0.73 to 0.99) and of CXR compared to usual care 1.01 (95% CI 0.87 to1.17) (Table 3). The estimated RRs were almost identical for the network meta-analysis sensitivity analysis (Table 3), with RR 0.85 (95% CI 0.76 to 0.97).

**Table 3:** Network meta-analysis, lung cancer mortality results

	**Relative risk (RR)**	**95% confidence interval**
Main analysis		
LDCT vs. usual care	0.86	0.75 to 0.98
LDCT vs. CXR	0.85	0.73 to 0.99
CXR vs. usual care	1.01	0.87 to 1.17
Sensitivity analysis		
LDCT vs. usual care	0.85	0.76 to 0.97
LDCT vs. CXR	0.86	0.75 to 0.98
CXR vs. usual care	0.99	0.89 to 1.12

For the main network meta-analysis, LDCT was ranked first with 97% probability, with usual care second (55%) or third (44%) and CXR second (43%) or third (56%) (Web Fig. 1).

**Fig. 4:**
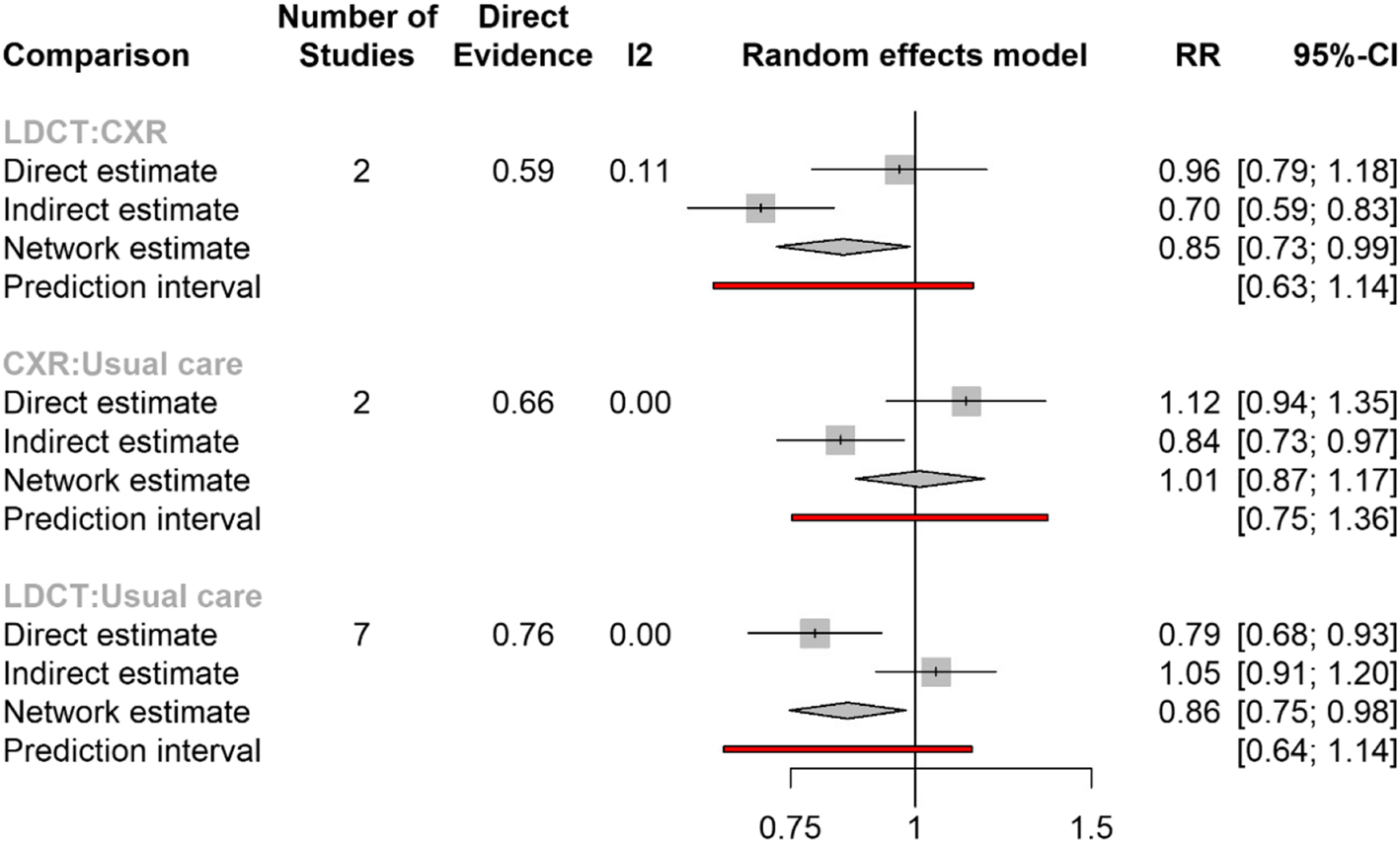
Network split—direct and indirect results (lung cancer mortality)

Direct and indirect results for the primary analysis are presented separately in Fig. 4. There is some inconsistency between direct and indirect results, as might be expected from the considerable heterogeneity in screening strategies employed in these trials. Despite this, the network results are not qualitatively different from the direct (pairwise) comparisons and the estimates from the two approaches are consistent with each other.

## Discussion

The main findings of the direct meta-analysis of RCTs comparing LDCT screening programmes with usual care (no screening) or other imaging screening programme (such as chest X-ray (CXR)) demonstrated a statistically significant decrease in lung cancer mortality (pooled RR 0.86, 95% CI 0.77 to 0.96, and a statistically non-significant decrease in all-cause mortality outcome (pooled RR 0.98, 95% CI 0.95 to1.01), with very little statistical heterogeneity for both outcomes. The risk of bias assessments did not modify these findings as studies were generally moderate to high quality. The single poorer-quality study made little or no difference to the pooled RR when removed from the meta-analysis in sensitivity analyses. The network meta-analysis is consistent with the direct meta-analysis concerning the size of the effect of LDCT on lung cancer mortality. It also indicates that this effect on lung cancer mortality is very similar irrespective of whether LDCT is compared with usual care (pooled RR 0.86, 95% CI 0.75 to 0.98) or with CXR (pooled RR 0.85, 95% CI 0.73 to 0.99).

These results represent a considerable change from the results of our original systematic review which found that LDCT screening with up to 9.80 years of follow-up was associated with a statistically non-significant reduction in lung cancer mortality compared with controls. This update provides much stronger evidence of a reduction in lung cancer mortality with additional trials and up to 12.3 years of follow-up. There is also much less statistical heterogeneity in this update despite considerable clinical heterogeneity. The results of the network meta-analysis suggest that effect of LDCT on lung cancer mortality is very similar irrespective of whether the comparator is usual care or CXR screening.

The contributors to the changes in results are firstly the increased numbers of included studies, with three additional RCTs of LDCT vs usual care (ITALUNG, LUSI and NELSON) and one additional RCT of LDCT vs CXR screening (LSS). However, more important in terms of numbers of added events are the updates to the results for MILD and NLST, particularly the latter. Updating the results for NLST from a median follow-up of 6.5 to 12.3 years generated 1584 events for lung cancer mortality and 6742 events for all-cause mortality. Combining the additional events from all other sources generated 748 events for lung cancer mortality and 2993 events for all-cause mortality (Web Table 5). Given this, it is important to note that there has been substantial change in the estimates of effect for NLST. The RR for lung cancer mortality was 0.80 (95% CI 0.70, 0.92) originally and 0.93 (95% CI 0.86, 1.00) in the update. The RR for all-cause mortality was 0.94 (95% CI 0.88, 1.00) originally and 0.98 (95% CI 0.95, 1.01) in the update.

Considering strengths and weaknesses, the research we report was undertaken by an experienced health technology assessment group, working to a pre-specified protocol, adhering to well-recognised standards for conducting systematic reviews. Further the research was an update, using the same method, as a highly scrutinised and multiply peer reviewed systematic review [12, 13]. No members of the research team had any connection with the trialists for the included RCTs. The research, both the original review and the update, was commissioned by the NIHR in the UK to inform the decision-making of the National Screening Committee, of which one author (CH) is a member. All key steps (screening search results, in/exclusion, data extraction and analysis) were undertaken by one member of the research team and checked by a second. The reporting conforms to PRISMA guidelines.

We did not have opportunity to systematically contact each of the original research teams which may have helped fill some of the gaps in details about the RCTs, particularly randomisation methods. We have searched for unpublished studies such as conference proceedings throughout both the original review and the update, thereby reducing the risk of publication bias. We did not formally examine for publication bias as the number of studies is not enough to get reliable results. The amount of available mortality data seems unlikely to grow greatly in the immediate future. Completed trials have reported at least 5 years follow-up and many around 10 years. This is however follow-up from randomisation, whereas similar periods of follow-up after completion of the intervention, such as has been achieved in NLST might arguably be the ideal. There are also other studies in progress: Yang et al. [28], Early Detection of Cancer of the Lung Scotland (ECLS) [26], and Yorkshire Lung Screening Trial (YLST) [29] (Web Table 2), so reviewing the mortality data from LDCT trials should continue. The results from Yang et al. in a lower-risk, Asian population will be of particular interest.

Our findings are consistent with recently published systematic reviews. Huang et al. reported a pooled RR of 0.83 (95% CI 0.76 to 0.90) for lung cancer mortality and 0.95 (95% CI 0.90 to 1.00) for all-cause mortality [64]. Hoffman et al. reported a pooled RR of 0.84 (95% CI 0.75 to 0.93) for lung cancer mortality and 0.96 (95% CI 0.91 to 1.01) for all-cause mortality [65]. Neither included the important long-term follow-up from NLST. The meta-analysis in Field et al. did include this and reported pooled RRs of 0.84 (95% CI 0.76 to 0.92) and 0.97 (95% CI 0.94 to 1.00) for lung cancer and all-cause mortality respectively [35]. The Cochrane review on this topic has recently been updated [66]. They offer more optimistic summary estimates, RR 0.79 (95% CI 0.72 to 0.87) for lung cancer mortality and RR 0.95 (95% CI 0.91 to 0.99) for all-cause mortality. Although they do include the long-term follow-up results from NLST in their review, they prefer data from “planned follow-up” for their headline analyses which is after 6.5 as opposed to 12.3 years post randomisation. Despite the growing number of systematic reviews on this topic, we suggest ours is of particular interest because it has tracked results as they have evolved and is the only one to use network meta-analysis to take the different nature of the comparators into account, by estimating relative effects on lung cancer mortality between different screening strategies. Unlike other reviews, we have not attempted to derive estimates of sex-specific estimates of effect and do not believe they are a useful addition to the evidence base.

There is considerable clinical heterogeneity between trials, including different frequency and number of screens and the use of a baseline screen in some of the control arms. There is some evidence of inconsistency between the direct and indirect evidence which is likely explained by these differences. This article only considers mortality, consistent with the original article. Now that there is confidence that the effect on mortality is beneficial, the wider balance between benefits and harms becomes important too, but is beyond the scope of this article. In addition, cost-effectiveness, which is currently highly uncertain [67], needs to be established, also taking this balance between benefits and harms into account. Our group, among others, is currently working on this. A particular challenge is taking learning about how to optimise the LDCT screening process into account. The low risk of events also needs to be considered. Here we note that the change in this update has also led to an improvement in the number needed to screen (NNS) to avoid one lung cancer death, from 357 (95% CI 82 to − 113)^1^ to 167 (95% CI 93 to 454). The assumed baseline risk is 4.64 lung cancer deaths per 100 persons over a 6-year period as found in DANTE, which identified the highest lung cancer risk of death in the RCTs contributing data on lung cancer mortality. Even with a lower NNS, a considerable number of participants still need to be screened multiple times over a period of at least 5 years to achieve one less lung cancer death even in high-risk populations. The use of risk assessment to tools to improve identification of those most at risk is actively under investigation, as is using information from initial screens to modify screening approach in subsequent rounds of screening.

## Conclusions

On balance, the evidence on mortality does now support implementation of LDCT in high-risk populations. The marked changes from our original review emphasise the importance of updating systematic reviews. There a number of RCTs unreported or in progress, but they are small relative to the total number of included participants in this review. There are unresolved issues, particularly the balance between benefits and harms overall and cost-effectiveness. Nonetheless, greater clarity on the presence and size of the effect on mortality should provide reassurance to the many countries who are currently still considering whether to introduce LDCT screening.

### Supplementary Information

**Additional file 1:** **Web Table 1.** Example search strategy for MEDLINE. **Web Table 2.** Included studies in qualitative systematic review. **Web Table 3.** Notes on the justification of risk of bias assessments. **Web Table 4.** Illustrating balance/imbalance in baseline characteristics between MILD and three other low dose CT RCTs included in direct meta-analysis. **Web Table 5.** Event data for included studies. **Web Figure 1.** Network meta-analysis rankogram – Main analysis.

## Data Availability

Data sharing not applicable to this article as no datasets were generated or analysed during the current study.

## Abbreviations

CI: Confidence interval
CT: Computed tomography
CXR: Chest X-ray
DANTE: Detection And screening of early lung cancer with Novel imaging Technology and molecular Essays
DLSCT: Danish Lung Cancer Screening Trial
ECLS: Early detection of Cancer of the Lung Scotland
ITALUNG: ITAlian LUNG cancer screening
LDCT: Low-dose computed tomography
LUSI: German LUng cancer Screening Intervention trial
MILD: Multicentric Italian Lung Detection project
NELSON: NEderlands Leuvens Longkanker Screenings ONderzoek
NLST: National Lung Screening Trial
NNS: Number needed to screen
PLCO: Prostate, Lung, Colorectal and Ovarian cancer screening trial
PRISMA: Preferred Reporting Items for Systematic reviews and Meta-Analyses
RCT: Randomised controlled trial
RR: Relative risk
UKLS: UK Lung Screening trial
YLTS: Yorkshire Lung Screening Trial

## Footnotes

1 negative value indicates screening increases lung cancer deaths; the confidence interval includes infinity, equivalent to a. risk reduction of 0, so the point estimate is encompassed by the interval although apparently lying outside it.
