# Supplementary material for: Do we know enough about the effect of low-dose computed tomography screening for lung cancer on mortality to act? An updated systematic review, meta-analysis and network meta-analysis of randomised controlled trials 2017 to 2021
Source: Diagn Progn Res. 2023 Dec 11;7:26. doi: 10.1186/s41512-023-00162-0 (PMC10712083; doi:10.1186/s41512-023-00162-0)

**WEB RESOURCES**

Web Table 1. Example search strategy for MEDLINE

Lung cancer screening searches – clinical effectiveness 1, 2004-Jan 2012, no comparator, low dose CT only, RCT filter

Database: MEDLINE

Host: Ovid

Data Parameters: 1946 to December week 1 2016

Date Searched: 9/1/2017

Searcher: SR

Hits: 183

1. exp Lung Neoplasms/

2. ((lung$ or bronch$ or pulmon$) adj3 (cancer$ or neopla$ or tumor$ or tumour$ or carcinoma$ or adenocarcinoma$ or small cell or squamous)).ti,ab,ot,kw.

3. (NSLC or NSCLC or SLC or SCLC).ti,ab,ot,kw.

4. 1 or 2 or 3

5. exp Tomography, X-Ray Computed/

6. ((CT or CAT) adj3 (scan$ or screen$)).ti,ab,ot,kw.

7. ((computer$ adj3 tomogra$) and (scan$ or screen$)).ti,ab,ot,kw.

8. (tomogra$ or helix or helical or spiral$ or spiro$).ti,ab,ot,kw.

9. 5 or 6 or 7 or 8

10. ((low$ adj3 dos$) or LDCT).ti,ab,kw,ot.

11. ((ultralow$ or ultra-low$) adj3 dos$).ti,ab,kw,ot.

12. (low-dos$ or ultralow-dos$).ti,ab,kw,ot.

13. 10 or 11 or 12

14. randomized controlled trial.pt.

15. controlled clinical trial.pt.

16. randomized.ab.

17. placebo.ab.

18. drug therapy.fs.

19. randomly.ab.

20. trial.ab.

21. groups.ab.

22. 11 or 12 or 13 or 14 or 15 or 16 or 17 or 18

23. exp animals/ not humans.sh.

24. 19 not 20

25. 4 and 9 and 13 and 24

26. (200407* or 200408* or 200409* or 200410* or 200411* or 200412* or 2005* or 2006* or 2007* or 2008* or 2009* or 2010* or 2011* or 201201*).ed.

27. 25 and 26

28. Limit 27 to English language and yr=”2004-“

Lung cancer screening searches – clinical effectiveness 2, 2012- current, all CT scan doses, X-ray comparator, RCT filter

Database: MEDLINE

Host: Ovid

Data Parameters: 1946 to December week 1 2016

Date Searched: 10/1/2017

Searcher: SR

Hits: 2074

1. exp Lung Neoplasms/

2. ((lung$ or bronch$ or pulmon$) adj3 (cancer$ or neopla$ or tumor$ or tumour$ or carcinoma$ or adenocarcinoma$ or small cell or squamous)).ti,ab,ot,kw.

3. (NSLC or NSCLC or SLC or SCLC).ti,ab,ot,kw.

4. 1 or 2 or 3

5. exp Tomography, X-Ray Computed/

6. exp Radiography, Thoracic/

7. (x ray or xray or x-ray or CXR or radiograph$).ti,ab,ot,kw.

8. ((CT or CAT) adj3 (scan$ or screen$)).ti,ab,ot,kw.

9. ((computer$ adj3 tomogra$) and (scan$ or screen$)).ti,ab,ot,kw.

10. (tomogra$ or helix or helical or spiral$ or spiro$).ti,ab,ot,kw.

11. 5 or 6 or 7 or 8 or 9 or 10

12. 4 and 11

13. (2012* or 2013* or 2014* or 2015* or 2016* or 2017*).ed.

14. randomized controlled trial.pt.

15. controlled clinical trial.pt.

16. randomized.ab.

17. placebo.ab.

18. drug therapy.fs.

19. randomly.ab.

20. trial.ab.

21. groups.ab.

22. 14 or 15 or 16 or 17 or 18 or 19 or 20 or 21

23. exp animals/ not humans.sh.

24. 22 not 23

25. 12 and 13 and 24

26. limit 25 to english language and yr=”2012-Current”

Web Table 2. Included studies in qualitative systematic review

| Study identifier & country | Recruitment time | Screening programme | Comparator | Sample size (n) | Age range, years | Population risk | Gender (% male) | Number of screening rounds | Screening times and interval (years) | Duration of follow-up (mean/  median) |
| --- | --- | --- | --- | --- | --- | --- | --- | --- | --- | --- |
| DANTE ^a^  Italy | March 2001 to February 2006 | LDCT, medical examination and one CXR | No screening, medical examination and one CXR | 2811 (2400 planned) | 60–74 | Smokers and ex-smokers | 100 | 5 | T0, T1, T2, T3, T4 (1-year intervals) | At December 2012, median 6 years 3.5 months |
| Depiscan ^b^  France | NR | LDCT | CXR | 765 | 47–76 (protocol 50–75) | Smokers and ex-smokers | 71 | 3 | T0, T1, T2 (1-year intervals) | NR |
| DLCST ^c^  Denmark | October 2004 to March 2006 | LDCT | No screening | 4104 | 50–70 | Smokers and ex-smokers | 55 | 5 | T0, T1, T2, T3, T4 (1-year intervals) | At April 2015, median: 9.80 years both arms |
| ECLS ^d^  UK | NR | Auto-antibodies blood test then CXR and LDCT | No screening | 12210 planned | 50-75 | Smokers and ex-smokers | NR  Adults recruited | 5 | T0, T0.5, T1, T1.5, T2 (6-monthly intervals) | At October 2019 NR (planned 2 years) |
| Garg et al. ^e^  USA | January 2001 to October 2001 | LDCT | No screening | 190 (400 planned) | 50–80 | Smokers and ex-smokers | 75 | 2 | T0, T1 (1-year interval) | NR. Feasibility study |
| Gaudio et ^f^ al. | NR | Cardiac CT + LDCT | Cardiac CT | Pilot  110 | 55-79 | Smokers and ex-smokers | 67 | 1 | T0 | At February 2019  Short term – feasibility study |
| ITALUNG ^g^  Italy | January 2004 to December 2006 | LDCT, smoking cessation programme | No screening, smoking cessation programme | 3206 | 55-59 | Smokers and ex-smokers | 65 | 4 | T0, T1, T2, T3 (1-year intervals) | At December 2014, median 9.3 years  At October 2019 (submission date), median 11.3 years |
| LSS-PLC ^h^  USA | Randomisation from September 2000 to November 2000 or January 2001 (depending on source) | LDCT | CXR | 3318 (3000 planned) | 55–74 | Smokers and ex-smokers | 59 | 2 | T0, T1 (1-year interval) | At December 2005, median 5.2 years both groups |
| LUSI ^i^  Germany | September 2007 to April 2011 | LDCT, smoking counselling | No screening, smoking counselling | 4052 (4000 planned) | 50–69 | Smokers and ex-smokers | 65 | 5 | T0, T1, T2, T3, T4 (1-year intervals) | At April 2018, mean 8.8 years overall |
| MILD ^j^  Italy | September 2005 to September 2011 | LDCT (annual and biannual), smoking cessation, pulmonary function test, blood sample | No screening, smoking cessation, pulmonary function test, blood sample | 4099 (10,000 planned) | > 49 | Smokers and ex-smokers | 66 | 3 or 6 | T0, T1, T2, T3, T4, T5, T6 (1-year intervals) vs. T0, T2, T4, T6 (2-year intervals) | At January 2011, median 4.4 years, both arms.  At June 2018, “10 year results” |
| NELSON ^k l^  The Netherlands & Belgium | Second half of 2003 to December 2006 | LDCT | No screening | 15,822 | 50–75 | Smokers and ex-smokers | 84 | 4 | T0, T1, T3, T5.5  (increas-ing intervals) | At December 2015, “minimum follow-up of 10 years” |
| NLST ^m n o^  USA | August 2002 to April 2004 | LDCT | CXR | 53,454 | 55–74 | Smokers and ex-smokers | 59 | 3 | T0, T1, T2 (1-year intervals) | At December 2009, median 6.5 years both groups.  At July 2018 (submission date), median 12.3 years |
| UKLS  ^p q^  UK | August 2011 to August 2012 | LDCT | No screening | 3968 (4000 planned) | 50–75 | Five-year lung cancer risk ≥ 5%, based on the LLPv2 | 75 | 1 | T0 | At September 2021: median 7.3 years |
| Yang et al.^r^  China | November 2013 to November 2014 | LDCT | No screening | 6717 randomised;  6657 baseline screening | 45-70 | Smokers, ex-smokers, family or personal history of cancer, occupational exposure, passive smoking, cooking oil fumes | 47 | 3 | T0, T2, T4 (2-year intervals) | At August 2017, “Followed for at least five years” (inconsistent with recruitment information) |
| YLST ^s^  UK | September 2018 to October 2022 | LDCT in high risk by Lung Health Check | No screening (no knowledge in RCT) | 6892 planned | 50-80 | Smokers and ex-smokers | NR  Adults recruited | NR | NR | At December 2019 NR (planned 6 years) |
| Abbreviations: DANTE, Detection and Screening of Early Lung Cancer with Novel Imaging Technology and Molecular Essays; DLCST, Danish Lung Cancer Screening Trial; ECLS, Early Detection of Cancer of the Lung Scotland; ITALUNG, Italian lung cancer screening; LLPv2 The Liverpool Lung Project lung cancer risk prediction algorithm version 2; LSS-PLCO, Lung Screening Study as part of the Prostate, Lung, Colorectal and Ovarian cancer screening trial; LUSI, German lung cancer screening intervention; MILD, Multicentric Italian Lung Detection NELSON; NEderlands Leuvens Longkanker Screenings ONderzoek NLST, National Lung Screening Trial; NR, not reported; UKLS, UK Lung Screening Trial; YLST, Yorkshire Lung Screening Trial. | | | | | | | | | | |
| References:  a Infante M, Cavuto S, Lutman FR, Brambilla G, Chiesa G, Ceresoli G, et al. A randomized study of lung cancer screening with spiral computed tomography: three-year results from the DANTE trial. Am J Respir Crit Care Med 2009;180(5):445-53.  b Blanchon T, Brechot JM, Grenier PA, Ferretti GR, Lemarie E, Milleron B, et al. Baseline results of the Depiscan study: a French randomized pilot trial of lung cancer screening comparing low dose CT scan (LDCT) and chest X-ray (CXR). Lung Cancer 2007;58(1):50-8.  c Pedersen JH, Ashraf H, Dirksen A, Bach K, Hansen H, Toennesen P, et al. The Danish randomized lung cancer CT screening trial--overall design and results of the prevalence round. J Thorac Oncol 2009;4(5):608-14.  d Sullivan F, Schembri S. PL02.03 Early Detection of Cancer of the Lung Scotland (ECLS): Trial Results. Journal of Thoracic Oncology 2019;14 (10 Supplement):S5.  e Garg K, Keith RL, Byers T, Kelly K, Kerzner AL, Lynch DA, et al. Randomized controlled trial with low-dose spiral CT for lung cancer screening: Feasibility study and preliminary results. Radiology 2002;225(2):506-10.  f Gaudio C, Tanzilli A, Mei M, Moretti A, Barilla F, Varveri A, et al. Concomitant screening of coronary artery disease and lung cancer with a new ultrafast-low-dose Computed Tomography protocol: A pilot randomised trial. Scientific reports 2019;9(1):13872  g Lopes Pegna A, Picozzi G, Falaschi F, Carrozzi L, Falchini M, Carozzi FM, et al. Four-year results of low-dose CT screening and nodule management in the ITALUNG trial. J Thorac Oncol 2013;8(7):866-75.  h Gohagan JK, Marcus PM, Fagerstrom RM, Pinsky PF, Kramer BS, Prorok PC, et al. Final results of the Lung Screening Study, a randomized feasibility study of spiral CT versus chest X-ray screening for lung cancer. Lung Cancer 2005;47(1):9-15.  i Becker N, Motsch E, Gross ML, Eigentopf A, Heussel CP, Dienemann H, et al. Randomized study on early detection of lung cancer with MSCT in Germany: study design and results of the first screening round. J Cancer Res Clin Oncol 2012;138(9):1475-86.  j Pastorino U, Rossi M, Rosato V, Marchianò A, Sverzellati N, Morosi C, et al. Annual or biennial CT screening versus observation in heavy smokers: 5-year results of the MILD trial. Eur J Cancer Prev 2012;21(3):308-15.  k Horeweg N, Scholten ET, de Jong PA, van der Aalst CM, Weenink C, Lammers JWJ, et al. Detection of lung cancer through low-dose CT screening (NELSON): A prespecified analysis of screening test performance and interval cancers. Lancet Oncol 2014;15(12):1341-50.  l Horeweg N, van der Aalst CM, Vliegenthart R, Zhao Y, Xie X, Scholten ET, et al. Volumetric computed tomography screening for lung cancer: three rounds of the NELSON trial. Eur Respir J 2013;42(6):1659-67.  m Aberle DR, Adams AM, Berg CD, Black WC, Clapp JD, Fagerstrom RM, et al. Reduced lung-cancer mortality with low-dose computed tomographic screening. N Engl J Med 2011;365(5):395-409.  n National Lung Screening Trial Research Team, Church TR, Black WC, Aberle DR, Berg CD, Clingan KL, et al. Results of initial low-dose computed tomographic screening for lung cancer. N Engl J Med 2013;368(21):1980-91.  o Gareen IF, Duan F, Greco EM, Snyder BS, Boiselle PM, Park ER, et al. Impact of lung cancer screening results on participant health-related quality of life and state anxiety in the National Lung Screening Trial. Cancer 2014;120(21):3401-9.  p Field JK, Duffy SW, Baldwin DR, Brain KE, Devaraj A, Eisen T, et al. The UK Lung Cancer Screening Trial: a pilot randomised controlled trial of low-dose computed tomography screening for the early detection of lung cancer. Health Technol Assess 2016;20(40):1-146.  q Field JK, Vulkan D, Davies MP, Baldwin DR, Brain KE, Devaraj A, et al. Lung cancer mortality reduction by LDCT screening: UKLS randomised trial results and international meta-analysis. The Lancet Regional Health-Europe 2021 Sep 11:100179.  r Yang W, Qian F, Teng J, Wang H, Manegold C, Pilz LR, et al. Community-based lung cancer screening with low-dose CT in China: Results of the baseline screening. Lung Cancer 2018;117:20-6.  s The Yorkshire Lung Screening Trial ISRCTN42704678 https://doi.org/10.1186/ISRCTN42704678 Accessed 1/6/2021 | | | | | | | | | | |

Web Table 3. Notes on the justification of risk of bias assessments

| DANTE ^a^ |
| --- |
| Random-sequence generation: ‘Subjects were randomised by a 1:1 scheme in blocks of four and stratified by centre according to a computer-generated list supplied by the data centre each week before the enrolment sessions’ ^a^  No information provided on allocation concealment.  No blinding but the assessment of mortality was not felt likely to be influenced by this.  Outcome assessment blinded: ‘A panel blinded to patient’s assignment reviewed the clinical cases whenever several competing causes of death were possible’ a  Loss to follow-up unlikely as ‘Life status data and death certificates were requested for the entire study population from local health registries’ ^a^  Outcome prespecified.  Met sample size target, but power calculation based on mortality reduction of 50%.  Baseline equivalence demonstrated with the exception of respiratory comorbidity: LDCT 35%, control 31%; p = 0.0321. |
| DLCST ^b^ |
| Random-sequence generation. ‘Participants were randomized by a computer program (random permuted blocks of 10 participants) to either annual screening by low-dose computed tomography (the screening group) or the control group, which was not offered CT screening’. ^b^  No information provided on allocation concealment.  No blinding, but the assessment of mortality was not felt likely to be influenced by this.  Outcome assessment blinded ‘An international independent death review board will be established’. ^b^  Loss to follow-up unlikely as Danish Civil Registration System checked annually.  Outcome prespecified.  Met target sample size (n = 4000). Designed to be adequately powered in combination with NELSON (n = 16,000) to detect 25% reduction in mortality at 10 years.  Baseline equivalence demonstrated. |
| ITALUNG ^c^ |
| Random sequence generation. “Eligible subjects only were centrally randomised by a software procedure in an active arm receiving annual low-dose CT for 4 years and a control arm receiving usual care but no screening.” ^c^  Allocation concealment achieved.  No blinding, but the assessment of mortality was not felt likely to be influenced by this.  Outcome assessment likely to be blinded because of use of cancer registry to detect deaths. ^c^  Loss to follow-up also unlikely because, “All randomised subjects will be followed up by cancer registry of the Tuscany Region (http://www.cspo.it/) for incidence and mortality.” ^c^  No mention of target sample size or power calculation. “However, our study was designed and planned with the perspective of pooling data with other RCTs in Europe and US contributing to the cooperative effort for the evaluation of the efficacy of low-dose CT lung cancer screening.” ^c^  Outcome prespecified.  Baseline equivalence achieved. ^c^ |
| LSS d e |
| Random sequence generation. “Once eligibility was established and consent was obtained, participants were randomized, using a secure web-based system that was maintained by the coordinating center, to one of the two study arms, LDCT scan or CXR. Randomization was stratified by age (in 5-year categories), sex, and screening center, using blocks of varying sizes.” ^d^  Allocation concealed.  No blinding, but the assessment of mortality was not felt likely to be influenced by this.  Outcome assessment blinded. “Several years after study completion, in 2007, a linkage analysis was performed linking LSS subjects to the National Death Index (NDI). For the linkage, participants’ personally identifiable information (PII) was utilized to link LSS subjects to the NDI, with all deaths from trial entry through 2005 included. The underlying cause of death variable was used to classify deaths into lung-cancer- or non-lung cancer-related.” ^e^  Loss to follow-up unlikely because of method of outcome assessment.  Unclear whether outcome was prespecified.  Met target sample size (n = 3000). Origin of this sample size not justified.  Baseline equivalence achieved. ^d^ |
| LUSI ^f g^ |
| Random sequence generation. “Afterward, electronic randomization was carried out using the randomization tool RANDI developed in the Biostatistic Branch of the DKFZ. The block randomization was stratified by age (\60 years vs. C60 years of age), gender, and smoking status (current vs former smoker).” ^f^  No information provided on allocation concealment.  No blinding, but the assessment of mortality was not felt likely to be influenced by this.  Outcome assessment blinded. “If lung cancer was mentioned in any way (n = 84), an end point committee composed of a chest surgeon (GF), two radiologists (MP, SD) and a pathologist (PAS) classified the cases using methods identical to those in NELSON, with full blinding with regard to the allocation of patients to either the screening or control arm.” ^g^  Loss to follow-up unlikely because, “The prospective ascertainment of mortality was principally based on record linkage with municipal population registers, to which we have electronic access and which provide information on vital status and date of death with almost daily actuality, and death certificates were retrieved from registers of the local health authorities.” ^g^  Outcome prespecified.  Met target sample size (n = 4000). Origin of this sample size not justified.  Baseline equivalence achieved. ^f g^ |
| MILD ^h^ |
| Random-sequence generation. No detail on method of randomisation. Control group referred to as ‘. . . observational control arm’ in the discussion section of main paper reporting results. ^h^  No blinding, but the assessment of mortality was not felt likely to be influenced by this.  Outcome assessment blinded ‘. . . Cancer Registry Office database of Lombardy which traced the vital status of all participants blindly, without knowing the random allocation’ ^h^  Loss to follow-up unlikely because of use of death and cancer registries.  Outcome prespecified.  Failed to meet sample target size (n = 10,000) which was powered to detect a 30% reduction in lung cancer mortality after 10 years. Single centre results rather than the originally planned multicentre study.  Pronounced imbalances in baseline characteristics in three important characteristics (sex, current smoking status and predicted FEV1). ^h^ |
| NELSON ^i^ |
| No details on methods of randomisation or allocation concealment found in main publications  No blinding, but the assessment of mortality was not felt likely to be influenced by this.  Outcome assessment blinded as national statistics were used. A validation study using a blinded expert panel preceded the decision to use national statistics as the source of the primary outcome data. ^i^  Loss to follow-up minimal, “Follow-up data were retrieved from national linkages  at approximately 5, 7, and 10 to 11 years of complete follow-up. A total of 18 persons (13  men and 5 women) could not be linked, because a digital consent form could not be retrieved.”  Outcome prespecified.” ^i^  The target sample size (n = 17,300) was substantially met. There was a detailed account of the power calculations.  Baseline equivalence achieved. ^i^ |
| NLST ^j k l^ |
| Random-sequence generation. ‘Randomization occurred after data co-ordinating centres confirmed that eligibility criteria had been met for a given individual; participants were then assigned to either the computerized tomography arm or chest radiograph arm in a 1 : 1 ratio, stratifying by site, sex, and 5-year age group. Stratified randomization was accomplished by use of a block size of six or eight, with block size chosen at random’. ^j^  Allocation concealment seems likely but not explicitly stated, thus categorised as unclear.  No blinding, but the assessment of mortality was not felt likely to be influenced by this.  Outcome assessment blinded ‘An endpoint verification team determined whether the cause of death was lung cancer . . . members of the team were not aware of the group assignments’. ^k^  Loss to follow-up unlikely because of use of National Death Index. ^l^  Outcome prespecified.  Met target sample size (n = 50,000). Powered to achieve a 20% reduction in lung cancer mortality.  Baseline equivalence demonstrated. |
| UKLS ^m n^ |
| Random sequence generation. ‘Following attendance at a research clinic, recruits were randomised by computer into the intervention arm (LDCT scan, screen group) or the control arm (usual care, non-screen group) at a ratio of 1:1’. ^m^  How allocation concealment was achieved is also detailed in this source.  No blinding, but the assessment of mortality was not felt likely to be influenced by this.  Outcome assessment blinded. “Outcomes from UK cancer and death registry data were provided by NHS Digital and the National Cancer Registration and Analysis Service (NCRAS) who were not aware of the participants’ allocated trial arm.” ^n^  Loss to follow-up unlikely because, “Health and mortality outcomes of UKLS participants in both study arms will be followed for 10 years, via the Office for National Statistics (ONS), the Hospital Episode Statistics database and the National Cancer Registration Service.” ^m^  Outcome prespecified.  Met target sample size (n = 4000). But powering unclear, “As this is a pilot trial, not powered for the end point of lung cancer mortality, analysis of the end point features is secondary in this plan, as the main UKLS was not funded. However, at the appropriate time, lung cancer mortality data will be analysed and pooled with the NELSON trial data” & “As the UKLS pilot is insufficiently powered to demonstrate a reduction in mortality, a further objective is to provide results for pooling with current European lung screening studies.” ^m^  Baseline equivalence achieved. ^m^ |
| References: |
| a Infante M, Cavuto S, Lutman FR, Brambilla G, Chiesa G, Ceresoli G, et al. A randomized study of  lung cancer screening with spiral computed tomography: three-year results from the DANTE trial.  Am J Respir Crit Care Med 2009;180:445–53. https://doi.org/10.1164/rccm.200901-0076OC |
| b Pedersen JH, Ashraf H, Dirksen A, Bach K, Hansen H, Toennesen P, et al. The Danish randomized  lung cancer CT screening trial – overall design and results of the prevalence round. J Thorac  Oncol 2009;4:608–14. https://doi.org/10.1097/JTO.0b013e3181a0d98f |
| c Lopes Pegna A, Picozzi G, Mascalchi M, Maria Carozzi F, Carrozzi L, Comin C, et al. Design,recruitment and baseline results of the ITALUNG trial for lung cancer screening with low-dose CT. Lung Cancer 2009;64:34–40. https://doi.org/10.1016/j.lungcan.2008.07.003 |
| d Gohagan JK, Marcus PM, Fagerstrom RM, Pinsky PF, Kramer BS, Prorok PC, et al. The Lung Screening Study: the National Cancer Institute’s randomized feasibility study of spiral CT versus chest X-ray in lung cancer screening. Chest 2004;126:114—21. |
| e Doroudi M, Pinsky PF, Marcus PM. Lung Cancer Mortality in the Lung Screening Study Feasibility Trial. JNCI Cancer Spectr. 2018;2:pky042. |
| f Becker N, Motsch E, Gross ML, Eigentopf A, Heussel CP, Dienemann H, et al. Randomized study on early detection of lung cancer with MSCT in Germany: study design and results of the first screening round. J Cancer Res Clin Oncol 2012;138(9):1475-86. |
| g Becker N, Motsch E, Trotter A, Heussel CP, Dienemann H, Schnabel PA, et al. Lung cancer mortality reduction by LDCT screening-Results from the randomized German LUSI trial. International Journal of Cancer 2020;146(6):1503-13. |
| h Pastorino U, Rossi M, Rosato V, Marchianò A, Sverzellati N, Morosi C, et al. Annual or biennial CT  screening versus observation in heavy smokers: 5-year results of the MILD trial. Eur J Cancer Prev  2012;21:308–15. https://doi.org/10.1097/CEJ.0b013e328351e1b6 |
| i de Koning HJ, van der Aalst CM, de Jong PA, Scholten ET, Nackaerts K, Heuvelmans MA, et al. Reduced Lung-Cancer Mortality with Volume CT Screening in a Randomized Trial. NEJM 2020;382(6):503-13. |
| j Aberle DR, Adams AM, Berg CD, Clapp JD, Clingan KL, Gareen IF, et al. Baseline characteristics of  participants in the randomized national lung screening trial. J Natl Cancer Inst 2010;102:1771–9.  https://doi.org/10.1093/jnci/djq434 |
| k Aberle DR, Adams AM, Berg CD, Black WC, Clapp JD, Fagerstrom RM, et al. Reduced lung-cancer  mortality with low-dose computed tomographic screening. N Engl J Med 2011;365:395–409.  https://doi.org/10.1056/NEJMoa1102873 |
| l National Center for Health Statistics. National Death Index. Hyattsville, MD: National Center for  Health Statistics. URL: www.cdc.gov/nchs/ndi/index.htm (accessed 14 June 2018). |
| m Field JK, Duffy SW, Baldwin DR, Brain KE, Devaraj A, Eisen T, et al. The UK Lung Cancer Screening  Trial: a pilot randomised controlled trial of low-dose computed tomography screening for the early  detection of lung cancer. Health Technol Assess 2016;20(40). https://doi.org/10.3310/hta20400 |
| n Field JK, Vulkan D, Davies MP, Baldwin DR, Brain KE, Devaraj A, et al. Lung cancer mortality reduction by LDCT screening: UKLS randomised trial results and international meta-analysis. The Lancet Regional Health-Europe 2021 Sep 11:100179. |

Web Table 4. Illustrating balance/imbalance in baseline characteristics between MILD and three other low dose CT RCTs included in direct meta-analysis

| **Study Characteristics** | **DANTE**^a^ | | **MAYO**^b^ | | **MILD**^c^ | | | **NELSON**^d^ | |
| --- | --- | --- | --- | --- | --- | --- | --- | --- | --- |
| N | 2450 | | 9211 | | 4099 | | | 15792 | |
| Trial arm | LDCT | Control | LDCT | Control | LDCT (biennial) | LDCT (annual) | Control | LDCT | Control |
| n (% of N) | 1264 (51.6) | 1186 (48.4) | 4618 (50.1) | 4593 (49.9) | 1186 (28.9) | 1190 (29.0) | 1723 (42.0) | 7900 (50) | 7892 (50) |
| Sex (% of n male) | NR | NR | 100 | 100 | 68.5 | 68.4 | 63.3 | 83.3 | 83.8 |
| Age (mean y) | 64.6 | 64.6 | 55.8^A^ | 55.7^A^ | 58.2^A^ | 58.3^A^ | 57.6^A^ | 58^B^ | 58^B^ |
| Occupational exposure (% of n) | 31.3 | 34.1 | 3.5 | 3.1 | NR | NR | NR | NR | NR |
| **Smoking** | | | | | | | | | |
| Current smokers (%) | 56.5 | 57.4 | 100 | 100 | 68.3 | 68.9 | 89.7 | 56.0 | 55.1 |
| Pack-years (mean) | 47.3 | 47.2 | 51.4 | 50.9 | 39^B^ | 39^B^ | 38^B^ | 38.0^B^ | 38.0^B^ |
| Smoking duration (mean y) | NR | NR | 36 | 36 | 38.4^A^ | 38.3^A^ | 38.5^A^ | 37.7^A^ | 37.5^A^ |
| Cigs/day (mean) | NR | NR | 29.4 | 28.4 | 26.3^A^ | 26.8^A^ | 25.2^A^ | 21.6^A^ | 21.7^A^ |
| Duration smoking cessation in former smokers (mean y) | NR | NR | NR | NR | NR | NR | NR | 4.5^A^ | 4.5^A^ |
| **Comorbidities** | | | | | | | | | |
| Respiratory | 35.3 | 31.2 | 14.8 | 14.4 | NR | NR | NR | NR | NR |
| Chronic bronchitis, emphysema or COPD | NR | NR | 7.3 | 7.5 | NR | NR | NR | NR | NR |
| Hypertension | 36.1 | 37.7 | NR | NR | NR | NR | NR | NR | NR |
| Cardiac | 12.6 | 13.9 | NR | NR | NR | NR | NR | NR | NR |
| Heart disease or heart attack | NR | NR | NR | NR | NR | NR | NR | NR | NR |
| Stroke | NR | NR | NR | NR | NR | NR | NR | NR | NR |
| PVD | 10.3 | 9.0 | NR | NR | NR | NR | NR | NR | NR |
| Diabetes | 8.3 | 8.4 | NR | NR | NR | NR | NR | NR | NR |
| Malignancies | NR | NR | NR | NR | NR | NR | NR | NR | NR |
| **Lung function** | | | | | | | | | |
| FEV1 (L) | NR | NR | NR | NR | NR | NR | NR | NR | NR |
| FEV1 <90% predicted | NR | NR | NR | NR | 27.7 | 28.2 | 19.2 | NR | NR |
| Other data available |  | |  | | Paper indicates that only “selected baseline characteristics” were reported | | |  | |

**Abbreviations:** NR, not reported; PVD, peripheral vascular disease; FEV, forced expiratory volume.

**Footnotes:** A, calculated by systematic review team; B, median rather than mean

**References:**

a Infante M, Cavuto S, Lutman FR, Passera E, Chiarenza M, Chiesa G, et al. Long-Term Follow-up Results of the DANTE Trial, a Randomized Study of Lung Cancer Screening with Spiral Computed Tomography. Am J Respir Crit Care Med 2015;191(10):1166-75.

b Marcus PM, Prorok PC. Reanalysis of the Mayo Lung Project data: the impact of confounding and effect modification. J Med Screen. 1999;6:47–9.

c Pastorino U, Rossi M, Rosato V, Marchianò A, Sverzellati N, Morosi C, et al. Annual or biennial CT screening versus observation in heavy smokers: 5-year results of the MILD trial. Eur J Cancer Prev 2012;21(3):308-15.

d de Koning HJ, van der Aalst CM, de Jong PA, Scholten ET, Nackaerts K, Heuvelmans MA, et al. Reduced Lung-Cancer Mortality with Volume CT Screening in a Randomized Trial. NEJM 2020;382(6):503-13. [Table S1 in appendix]

Web Table 5. Event data for included studies

| **Study** | **Comparator** | **Lung cancer mortality** | | | | **All-cause mortality** | | | |
| --- | --- | --- | --- | --- | --- | --- | --- | --- | --- |
|  |  | Number of events | Total number of participants | Number of events | Total number of participants | Number of events | Total number of participants | Number of events | Total number of participants |
|  |  | Low dose CT group | | Control group | | Low dose CT group | | Control group | |
| Original systematic review up to 2017 | | | | | | | | | |
| DANTE^a^ | Usual care | 59 | 1264 | 55 | 1186 | 180 | 1264 | 176 | 1186 |
| DLCST^b^ | Usual care | 39 | 2052 | 38 | 2052 | 165 | 2052 | 163 | 2052 |
| MILD^c^ (Annual) | Usual care | 12 | 1190 | 7 | 1723 | 31 | 1190 | 20 | 1723 |
| MILD^c^ (Biannual) | Usual care | 6 | 1186 |  |  | 20 | 1186 |  |  |
| NLST^d^ | CXR | 356 | 26722 | 443 | 26732 | 1877 | 26722 | 2000 | 26732 |
|  |  |  |  |  |  |  |  |  |  |
|  |  | CXR group | | Control group | |  | |  | |
| Czech^e^ | Single CXR | 247 | 3172 | 216 | 3174 |  |  |  |  |
| MAYO^f^ | Usual care | 337 | 4607 | 303 | 4585 |  |  |  |  |
| PLCO^g^ (NLST eligible sub-group) | No screening | 316 | 15183 | 334 | 15138 |  |  |  |  |
|  |  |  |  |  |  |  |  |  |  |
| Updated systematic review up to 2021 | | | | | | | | | |
|  |  | Low dose CT group | | Control group | | Low dose CT group | | Control group | |
| DANTE^a^ | Usual care | 59 | 1264 | 55 | 1186 | 180 | 1264 | 176 | 1186 |
| DLCST^b^ | Usual care | 39 | 2052 | 38 | 2052 | 165 | 2052 | 163 | 2052 |
| ITALUNG ^h^ | Usual care | 58 | 1613 | 74 | 1593 | 154 | 1613 | 181 | 1593 |
| LSS ^i^ | CXR | 32 | 1660 | 26 | 1658 | 139 | 1660 | 116 | 1658 |
| LUSI ^j^ | Usual care | 29 | 2029 | 40 | 2023 | 148 | 2029 | 150 | 2023 |
| NELSON ^k^ | Usual care | 186 | 7895 | 248 | 7879 | 959 | 7895 | 974 | 7879 |
| MILD ^l^ (Annual) | Usual care | 19 | 1190 | 40 | 1723 | 76 | 1190 | 106 | 1723 |
| MILD ^l^ (Biannual) | Usual care | 21 | 1186 |  |  | 61 | 1186 |  |  |
| NLST ^m^ | CXR | 1147 | 26722 | 1236 | 26730 | 5253 | 26722 | 5366 | 26730 |

| UKLS ^n^ | Usual care | 30 | 1987 | 46 | 1981 | 246 | 1987 | 266 | 1981 |
| --- | --- | --- | --- | --- | --- | --- | --- | --- | --- |

|  |  | CXR group | | Control group | |  | |  | |
| --- | --- | --- | --- | --- | --- | --- | --- | --- | --- |
| Czech^e^ | Single CXR | 247 | 3172 | 216 | 3174 |  |  |  |  |
| MAYO^f^ | Usual care | 337 | 4607 | 303 | 4585 |  |  |  |  |
| PLCO^g^ (NLST eligible sub-group) | No screening | 316 | 15183 | 334 | 15138 |  |  |  |  |
| **Notes:**  Underlined entries indicate changes from original meta-analysis  **References:** | | | | | | | | | |
| a Infante M, Cavuto S, Lutman FR, Brambilla G, Chiesa G, Ceresoli G, et al. A randomized study of lung cancer screening with spiral computed tomography: three-year results from the DANTE trial. Am J Respir Crit Care Med 2009;180(5):445-53. | | | | | | | | | |
| b Pedersen JH, Ashraf H, Dirksen A, Bach K, Hansen H, Toennesen P, et al. The Danish randomized lung cancer CT screening trial--overall design and results of the prevalence round. J Thorac Oncol 2009;4(5):608-14. | | | | | | | | | |
| c Pastorino U, Rossi M, Rosato V, Marchianò A, Sverzellati N, Morosi C, et al. Annual or biennial CT screening versus observation in heavy smokers: 5-year results of the MILD trial. Eur J Cancer Prev 2012;21(3):308-15. | | | | | | | | | |
| d National Lung Screening Trial Research Team, Aberle DR, Adams AM, Berg CD, Black WC, Clapp JD et al. Reduced lung-cancer mortality with low-dose computed tomographic screening. N Engl J Med 2011;365(5):395-409. | | | | | | | | | |
| e Kubık AK, Parkin DM, Zatloukal P. Czech Study on Lung Cancer Screening. Post-trial follow-up of lung cancer deaths up to year 15 since enrollment. Cancer 2000;89:2363–8. | | | | | | | | | |
| f Marcus PM, Bergstralh EJ, Fagerstrom RM, Williams DE, Fontana R, Taylor WF, et al. Lung cancer mortality in the Mayo Lung Project: Impact of extended follow-up. J Natl Cancer Inst 2000;92(16):1308-16. | | | | | | | | | |
| g Oken MM, Hocking WG, Kvale PA, Andriole GL, Buys SS, Church TR, et al. Screening by chest radiograph and lung cancer mortality: the Prostate, Lung, Colorectal, and Ovarian (PLCO) randomized trial. JAMA 2011;306(17):1865-73.  h Paci E, Puliti D, Carozzi FM, Carrozzi L, Falaschi F, Pegna AL, et al. Prognostic selection and long-term survival analysis to assess overdiagnosis risk in lung cancer screening randomized trials. J Med Screen 2021;28(1):39-47. Epub May 2020.  i Doroudi M, Pinsky PF, Marcus PM. Lung Cancer Mortality in the Lung Screening Study Feasibility Trial. JNCI Cancer Spectr. 2018;2:pky042.  j Becker N, Motsch E, Trotter A, Heussel CP, Dienemann H, Schnabel PA, et al. Lung cancer mortality reduction by LDCT screening-Results from the randomized German LUSI trial. International Journal of Cancer 2020;146(6):1503-13.  k de Koning HJ, van der Aalst CM, de Jong PA, Scholten ET, Nackaerts K, Heuvelmans MA, et al. Reduced Lung-Cancer Mortality with Volume CT Screening in a Randomized Trial. NEJM 2020;382(6):503-13.  l Pastorino U, Silva M, Sestini S, Sabia F, Boeri M, Cantarutti A, et al. Prolonged Lung Cancer Screening Reduced 10-year Mortality in the MILD Trial. Ann Oncol 2019; 30: 1162–1169.  m Aberle DR, Black WC, Chiles C, Church TR, Gareen IF, Gierada DS, et al. Lung Cancer Incidence and Mortality with Extended Follow-up in the National Lung Screening Trial. Journal of Thoracic Oncology. 2019;14(10):1732-42.  n Field JK, Vulkan D, Davies MP, Baldwin DR, Brain KE, Devaraj A, et al. Lung cancer mortality reduction by LDCT screening: UKLS randomised trial results and international meta-analysis. The Lancet Regional Health-Europe 2021;Sep 11:100179. | | | | | | | | | |

Web Figure 1. Network meta-analysis rankogram – Main analysis


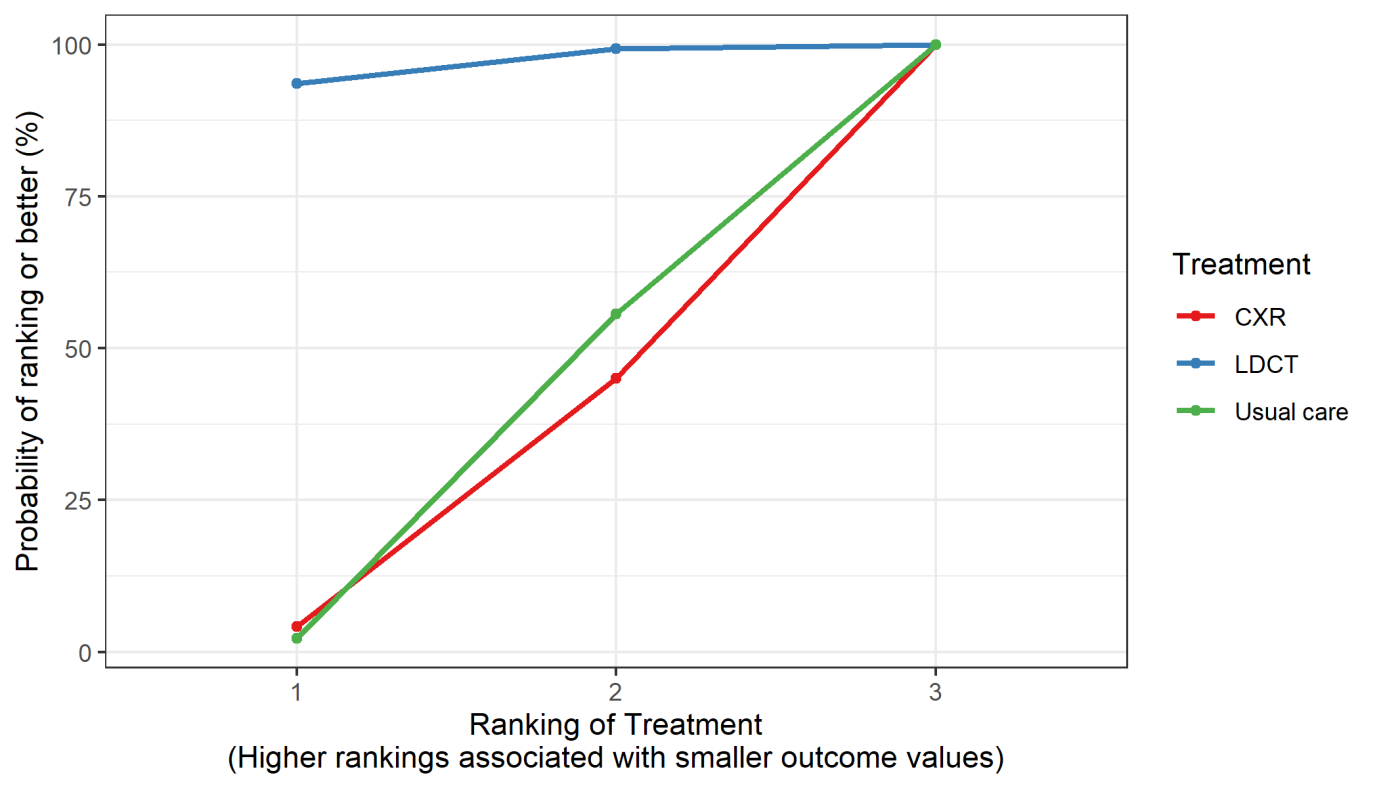

Supplement: Supplementary file 1 — Additional file 1: Web Table 1. Example search strategy for MEDLINE. Web Table 2. Included studies in qualitative systematic review. Web Table 3. Notes on the justification of risk of bias assessments. Web Table 4. Illustrating balance/imbalance in baseline characteristics between MILD and three other low dose CT RCTs included in direct meta-analysis. Web Table 5. Event data for included studies. Web Figure 1. Network meta-analysis rankogram – Main analysis. [file 41512_2023_162_MOESM1_ESM.docx]
